# CpG site degeneration triggered by the loss of functional constraint created a highly polymorphic macaque drug-metabolizing gene, *CYP1A2*

**DOI:** 10.1186/1471-2148-11-283

**Published:** 2011-10-01

**Authors:** Yasuhiro Uno, Naoki Osada

**Affiliations:** 1 Pharmacokinetics and Bioanalysis Center, Shin Nippon Biomedical Laboratories, Ltd., Kainan, Wakayama 642-0017, Japan; 2 Department of Population Genetics, National Institute of Genetics, 1111 Yata, Mishima, Shizuoka 411-8540, Japan; 3 Department of Genetics, The Graduate University for Advanced Studies (SOKENDAI), 1111, Yata, Mishima, Shizuoka 411-8540, Japan

## Abstract

**Background:**

Elucidating the pattern of evolutionary changes in drug-metabolizing genes is an important subject not only for evolutionary but for biomedical research. We investigated the pattern of divergence and polymorphisms of macaque *CYP1A1 *and *CYP1A2 *genes, which are major drug-metabolizing genes in humans. In humans, *CYP1A2 *is specifically expressed in livers while *CYP1A1 *has a wider gene expression pattern in extrahepatic tissues. In contrast, macaque *CYP1A2 *is expressed at a much lower level than *CYP1A1 *in livers. Interestingly, a previous study has shown that *Macaca fascicularis CYP1A2 *harbored unusually high genetic diversity within species. Genomic regions showing high genetic diversity within species is occasionally interpreted as a result of balancing selection, where natural selection maintains highly diverged alleles with different functions. Nevertheless many other forces could create such signatures.

**Results:**

We found that the *CYP1A1/2 *gene copy number and orientation has been highly conserved among mammalian genomes. The signature of gene conversion between *CYP1A1 *and *CYP1A2 *was detected, but the last gene conversion event in the simian primate lineage occurred before the *Catarrhini-Platyrrhini *divergence. The high genetic diversity of macaque *CYP1A2 *therefore cannot be explained by gene conversion between *CYP1A1 *and *CYP1A2*. By surveying *CYP1A2 *polymorphisms in total 91 *M. fascicularis *and *M. mulatta*, we found several null alleles segregating in these species, indicating functional constraint on *CYP1A2 *in macaques may have weakened after the divergence between humans and macaques. We propose that the high genetic diversity in macaque *CYP1A2 *is partly due to the degeneration of CpG sites, which had been maintained at a high level by purifying selection, and the rapid degeneration process was initiated by the loss of functional constraint on macaque *CYP1A2*.

**Conclusions:**

Our findings show that the highly polymorphic *CYP1A2 *gene in macaques has not been created by balancing selection but by the burst of CpG site degeneration after loss of functional constraint. Because the functional importance of *CYP1A1/2 *genes is different between humans and macaques, we have to be cautious in extrapolating a drug-testing data using substrates metabolized by *CYP1A *genes from macaques to humans, despite of their somewhat overlapping substrate specificity.

## Background

Cytochrome P450 oxidase (CYP) proteins oxidize a wide variety of substrates. They could metabolize not only many endogenous substrates such as steroid hormones, but also exogenous toxins for excretion [1]. In biomedical research, CYP are widely recognized as drug-metabolizing enzymes. Probably, normal function of drug-metabolizing CYPs in natural habitats would be detoxication of xenophobic chemicals taken from foods, especially from plants [2].

Humans have about 60 *CYP *genes in the genome [3]. Among them, *CYP1A *gene subfamily is one of the most major *CYP *subfamilies in humans. The human genome contains two *CYP1A *genes on chromosome 15, *CYP1A1 *and *CYP1A2*, which are tandemly aligned with 23-kb interval and head-to-head orientation [4]. The amino acid sequence identity between the paralogs is > 80% and their protein structures are supposed to be very similar, partly accounting for overlapping substrate selectivity of these enzymes [5]. Human *CYP1A2 *is constitutively expressed in livers and contributes to the hepatic metabolism of many important chemical compounds such as caffeine [6]. In contrast, the tissue distribution of human *CYP1A1 *gene expression is very broad and its expression is strongly induced by exogenous compounds, indicating *CYP1A2 *rather than *CYP1A1 *is a major hepatic *CYP1A *gene in humans. Polymorphisms in human *CYP1A *genes have been intensively studied for medical benefits (e.g., [7]), while a few studies from an evolutionary standpoint have been conducted for human *CYP1A *genes [8,9]. For example, Jorge-Nebert et al. surveyed the pattern of single nucleotide polymorphisms (SNPs) in several human populations at the *CYP1A1-CYP1A2 *locus and found the signature of selective sweep around the *CYP1A1 *untranslated region [9]. However, the role of the selective sweep for human *CYP1A1 *evolution remains unclear.

Because macaque monkeys are widely used for testing drug toxicity in preclinical trials, the genetic similarity of *CYP *genes between humans and macaques is important not only for evolutionary research but also for biomedical applications [10]. Unexpected phenotypic difference between divergent species is one the biggest concerns in drug development, e.g., [11]. A previous study has suggested that drug metabolism by CYP1A1/2 was not perfectly conserved among humans, macaques, and marmosets [12]. Elucidating the cause of phenotypic differences among primates at a genetic level is an important task.

Our previous sequence analysis showed that the region encompassing the first intron and exon 2 of macaque *CYP1A2 *had the highest genetic diversities among randomly selected 54 unlinked autosomal loci [13]. Because allelic variation of CYP1A2 proteins may increase a repertory of toxic substrates metabolized by CYP1A2, it is reasonable to imagine that the high genetic diversity in macaque CYP1A2 has been maintained by balancing selection, where natural selection maintains highly diverged alleles having different functions [14,15]. However, other studies also have shown that the gene expression pattern of *CYP1A2 *in macaques was quite different from that in humans. The level of gene expression of macaque *CYP1A2 *is very low in all *Macaca fascicularis *(cynomolgus macaques) livers so far examined [16,17]. The expression level of *CYP1A1 *is instead much more abundant in macaque livers. Interestingly, the pattern of CYP1A1/2 protein expression appears similar between humans and marmosets [12], indicating a major hepatic *CYP1A *gene was switched at some time after the divergence of the human and macaque lineages [17]. The result casts doubt that the balancing selection hypothesis accounts for high *CYP1A2 *diversity in macaques. However, if macaque *CYP1A2 *lost its function, the evolutionary history of *CYP1A2 *should resemble many other neutral loci.

In order to answer the question why macaque *CYP1A2 *has unusually high genetic diversity, we first investigated the long-term evolution pattern of *CYP1A *subfamily. Gene conversion between paralogs was examined with particular attention, because gene conversion between paralogs may create an unusual pattern of polymorphisms in paralogous genes. We found that the last gene conversion event between *CYP1A1 *and *CYP1A2 *in the lineages to *Catarrhini *primates occurred after the divergence of primates and rodents, and before the divergence of *Catarrhini *and *Platyrrhini *primates. We further sequenced *CYP1A1/2 *coding regions of 63 *M. fascicularis *and 28 *M. mulatta *individuals, and found several deletions and single nucleotide polymorphisms in macaque *CYP1A2 *with relatively high frequencies, which cause premature stop codons. We propose that macaque *CYP1A2 *is in the process of pseudogenization, and rapid degeneration of CpG sites is creating a high genetic diversity.

## Results

### Phylogenetic analysis of mammalian *CYP1A *subfamily

**Figure 1:**
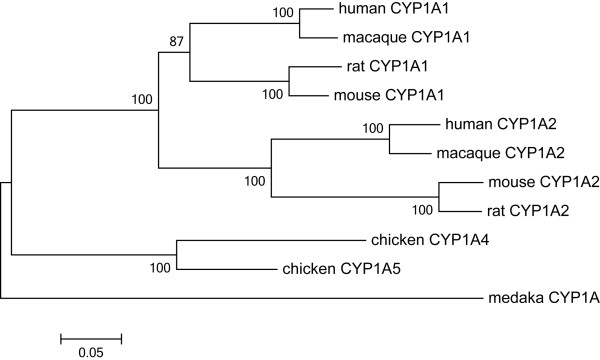
**Phylogenetic tree of vertebrate *CYP1A *subfamily genes**. Amino acid sequences of entire proteins were used for the tree reconstruction. The bootstrap values (%) are shown on the branches.

We first analyzed the general pattern of molecular evolution in the mammalian *CYP1A *subfamily. The phylogenetic tree of *CYP1A1/2 *genes was constructed using the cDNA sequences registered in the public database (Figure 1). Although two independent gene duplications were assigned on the phylogenetic tree, Goldstone and Stegeman [18] have suggested that intense inter-locus gene conversion occurred in the lineage of chickens; i.e., mammalian *CYP1A1/2 *genes and chicken *CYP1A5*/4 genes are orthologous, respectively. We further searched syntenic regions to the human *CYP1A1-CYP1A2 *locus from the draft genome sequences of several vertebrate genomes. We found that teleost fish (medaka) and amphibian (xenopus) genomes contained only one *CYP1A *gene, while chicken, opossum, and other placental mammalian genomes (human, orangutan, macaque, mouse, rat, rabbit, guinea pig, cow, horse, dog, and elephant) encompassed two genes in a head-to-head orientation. In addition, a previous study has shown that all fish *CYP1A *genes formed a monophyletic cluster [5]. Therefore, gene duplication of *CYP1A1/2 *was supposed to occur at some time between the Amphibia-Amniota divergence and Mammalia-Sauropsida divergence, around 300-350 Mya [19,20], and the number of genes has been stable since the gene duplication.

We also investigated whether macaques carry additional copy of *CYP1A *genes other than *CYP1A1/2 *in the genome. The latest draft genome sequence of *M. mulatta *(rheMac2) did not contain any other sequence similar to *CYP1A*. Furthermore, a previous search for genes expressed in *M. fascicularis *livers did not identify any additional macaque *CYP1A *paralogs [21], suggesting that *CYP1A1 *and *CYP1A2 *are the only *CYP1A *genes in macaque genomes.

### Gene conversion between *CYP1A1 *and *CYP1A2*

The pattern of paralogous divergence between *CYP1A1 *and *CYP1A2 *genes was further investigated. In order to grasp an overall picture, we first focused on only human and mouse genes. Amino acid differences (*p*-distance) between human and mouse orthologs and between paralogs for each coding exon are summarized in Table 1. Considering that the gene duplication of *CYP1A1/2 *occurred more than 300 Mya and the divergence between primates and rodents occurred around 80-120 Mya, paralogous genes should have much more diverged than human-mouse orthologs. Nevertheless, in exon 2 and 5, the average level of protein divergence between paralogous genes was smaller than that between human-mouse orthologs. The pattern suggests that gene conversion between *CYP1A1 *and *CYP1A2 *may have homogenized the paralogous gene sequences after the divergence of primates and rodents.

**Table 1:** Amino acid *p*-distance between human-mouse CYP1A1/2 quartets

	Exon2	Exon3	Exon4	Exon5	Exon6	Exon7
*hCYP1A1-mCYP1A1*	0.222	0.167	0.034	0.103	0.107	0.266
*hCYP1A2-mCYP1A2*	0.289	0.263	0.310	0.179	0.250	0.253
*hCYP1A1-hCYP1A2*	0.216	0.569	0.172	0.128	0.179	0.309
*mCYP1A1-mCYP1A2*	0.215	0.655	0.241	0.051	0.214	0.372

*h and *m *represent human and mouse genes, respectively.

**Figure 2:**
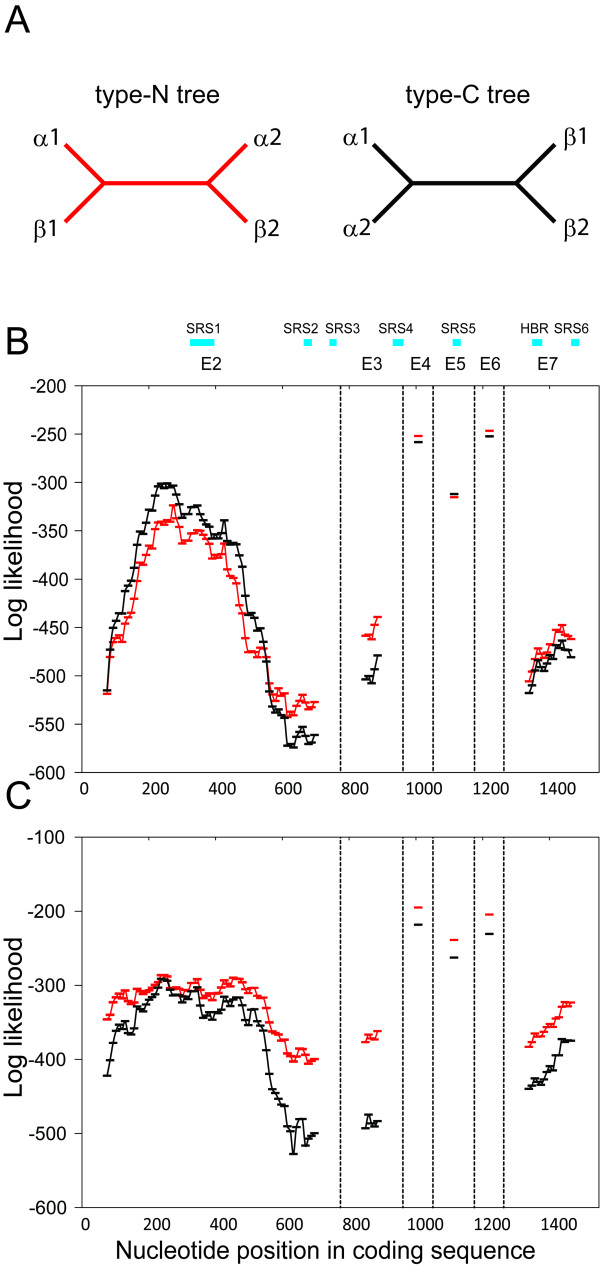
**Test of region-specific gene conversion**. Gene duplication occurred before the divergence of two species (α and β) and each species has two copies of genes (1 and 2). A) When gene conversion occurred after the divergence of two species, the tree topology would become the type-C tree (right); otherwise the tree would become the type-N tree (left). B) The log-likelihood values of the type-N (red lines) and type-C trees (black lines) in the human-mouse quartet genes. Substrate recognition sites (SRS) and heme-binding regions (HBR) are shown as the blue squares. The vertical dashed lines represent boundaries between exons. C) The log-likelihood values in the human-marmoset quartet genes.

In order to detect gene conversion between *CYP1A1 *and *CYP1A2*, we analyzed the human-mouse quartet gene set using a maximum likelihood framework. Figure 2A shows the expected patterns of paralogous gene divergence in two species when the gene duplication preceded the split of species. If gene conversion between paralogs does not occur, a type-N tree in Figure 2A would be observed. On the other hand, if gene conversion homogenized the two paralogous sequences, the phylogenetic pattern among the quartet genes would become a type-C tree in Figure 2A. Since the two types of trees are unrooted trees, the topology of trees with gene conversion only in one species would become the same as the type-C tree. In order to examine the regional heterogeneity of divergence pattern, we binned exon 2, 3, and 7 into 150-bp-length windows with 9-bp (3 codons) sliding steps; exon 4, 5, and 6 were not subdivided because they were shorter than 150 bp. For each window, likelihood values of the type-N and type-C trees were maximized using PAML [22]. If gene conversion occurred after the divergence of primates and rodents, those regions would have a higher probability of the type-C tree than that of the type-N tree. Between humans and mice, the first 500 bp of exon 2 and entire exon 5 had higher likelihood values of the type-C tree than that of the type-N tree (Figure 2B), indicating gene conversion had homogenized these regions after the divergence of primates and rodents. The highest log-likelihood ratio was 39.3 in the window at 237 bp, which corresponded to *P *< 10^-22^. The regions under gene conversion encompassed substrate recognition site (SRS) 1 and 5. We repeated the analysis using a human-marmoset quartet set. Interestingly, the high-probability regions of the type-C tree were not observed in the human-marmoset quartets (Figure 2C), suggesting that gene conversion did not occur in the *Catarrhini *lineage after the divergence of *Catarrhini *and *Platyrrhini*.

### Genetic diversity of *CYP1 *genes in *M. fascicularis*

Since gene conversion could not explain the high genetic diversity of macaque *CYP1A2*, detailed analyses on macaque *CYP1A1/2 *polymorphisms were conducted. We determined complete coding sequences of *CYP1A1 *and *CYP1A2 *genes of 10 and 7 *M. fascicularis *individuals, respectively, by sequencing PCR amplicons of genomic DNA. Basic population genetic statistics such as nucleotide diversity (*π*), number of segregating sites (*S*), and Tajima's *D *are summarized in Table 2. *CYP1A1 *showed less genetic diversity than *CYP1A2 *both at synonymous and nonsynonymous sites. Both genes showed similar level of negative Tajima's *D *values as those in other loci studied in the previous study (average was -0.68 with 0.64 standard deviation in 27 randomly selected genes [13]). Although negative *D *statistics might have been caused by selective force around the locus such as selective sweep, the prevalence of negative *D *statistics in the genome suggested the past population expansion of *M. fascicularis *and *M. mulatta*.

**Table 2:** Genetic diversity of *M.fascicularis CYP1A1/2 *genes

	*N*	*S*	*π*	*π*_S_	*π*_A_	*D*
*CYP1A1*	20	7	0.00072	0.00219	0.00026	-1.446
*CYP1A2*	14	31	0.00462	0.00740	0.00375	-1.139

*N, number of sampled chromosomes; *S*, number of segregating sites; *π*, nucleotide diversity, at synonymous sites (*π*_S_) and nonsynonymous sites (*π*_A_); *D*, Tajima's *D *statistics

In those samples, four null mutations were segregating in *M. fascicularis CYP1A2*. If the function of *CYP1A2 *were completely lost before the most recent common ancestor (MRCA) of macaques, mutations would be randomly segregating at nonsynonymous and synonymous sites and the ratio of per-site nucleotide diversity at nonsynonymous site to synonymous sites (*π*_A_/*π*_S_) would be close to 1. The ratio of nonsynonymous nucleotide diversity (*π*_A_) to synonymous nucleotide diversity (*π*_S_) in *CYP1A2 *was 0.51, much smaller than 1, although larger than the ratio in *CYP1A1 *(*π*_A_/*π*_S _= 0.12). The ratio smaller than 1 indicates that the *CYP1A2 *gene in macaques lost its function after the occurrence of MRCA, or the function of *CYP1A2 *is partly retained: i.e., null alleles have been weakly selected against in macaque *CYP1A2*. When we analyzed only individuals that have both *CYP1A1 *and *CYP1A2 *sequences (12 chromosomes), the estimated values of *π *were similar to the values with all samples (0.00075 and 0.00442 in *M. fascicularis CYP1A1 *and *CYP1A2*, respectively).

In order to investigate whether the different pattern of genetic diversity between *CYP1A1 *and *CYP1A2 *genes are simply due to difference in functional constraint and/or mutation rates, HKA tests were conducted using human orthologous genes as outgroup genes [23]. *M. fascicularis *individuals having both *CYP1A1 *and *CYP1A2 *sequences were analyzed and the test was significant (*P *= 0.0415), indicating macaque *CYP1A2 *has relatively high polymorphism given the level of divergence. The marginal significance in *M. fascicularis *was improved (*P *= 0.0224) when an additional eight *M. mulatta *chromosomes were analyzed together.

### Resequencing of a large number of *M. fascicularis *and *M. mulatta CYP1A2*

To elucidate the mechanisms for creating polymorphisms in macaque *CYP1A2*, we sequenced the first 1166 bp of the coding sequences, which spanned from exon 2 to exon 4, of additional 63 *M. fascicularis *and 28 *M. mulatta *samples. We identified five null mutations in those samples (Table 3). Among those individuals, 38 and 7 macaques carried null alleles in the coding regions as heterozygotes and homozygotes, respectively. For the null mutations of high frequency at site 1066, Hardy-Weinberg equilibrium was examined. In both *M. fascicularis *and *M. mulatta*, the deviation from the equilibrium was not detected (*P *= 0.874 and *P *= 0.753, respectively; chi-square test). The prevalence of null alleles and homozygous individuals supports that *CYP1A2 *is a dispensable gene in macaques and the effect of purifying selection on macaque *CYP1A2 *is considerably weak. Among 65 SNPs in this region, 36 were *M. fascicularis *specific, 16 were *M. mulatta *specific, and 13 were shared between species. No fixed difference between species was discovered.

**Table 3:** Allele frequency of null alleles in macaque *CYP1A2*

Position in coding sequence	Type	*M. fascicularis*	*M. mulatta*
138	G to A	0/126	5/51
286	1 bp deletion	4/122	0/56
1066	C to T	8/118	22/34
1090	C to T	2/124	0/56
1103	T to A	11/115	0/56

*The number of null alleles is shown in the left of slash (/).

Interestingly, 32 of 65 SNPs were at CpG dinucleotide sites. It is well known that methylated dinucleotide CG tends to mutate to CA and TG in mammalian genomes, probably at > 10 times faster than the background mutation rate [24,25]. In agreement with this prediction, 30 of 32 mutations at CpG sites were mutations from CG to CA or from CG to TG, assuming human *CYP1A2 *had ancestral alleles. Among those mutations, 20, 9, and 1 were synonymous, nonsynonymous, and null mutations, respectively. We counted the number of CpG dinucleotides in the reference *M. fascicularis CYP1A1/2 *cDNA sequences (*CYP1A1*, D17575; *CYP1A2*, D86474). Macaque *CYP1A1 *and *CYP1A2 *had 41 and 63 CpG dinucleotides in the protein coding regions, respectively; the macaque *CYP1A2 *contained CpG sites about 8% of the coding sequence. Among all annotated genes in the *M. mulatta *genome sequence (RefSeq genes, [26]), the fraction of CpG sites in *CYP1A2 *was ranked in the upper 8.3%.

**Figure 3:**
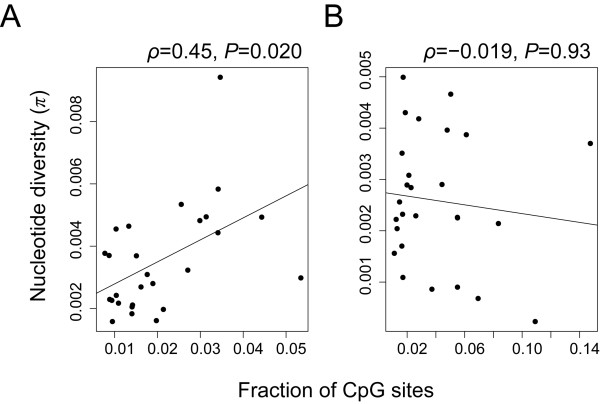
**Fraction of CpG sites and nucleotide diversity in *M. fascicularis***. The data is obtained from Osada et al. [13]. Liner regression lines are represented as the solid lines. Note that the correlation test was performed using a non-parametric test (Spearman's rank correlation test). A) Intergenic regions that are away from at least 100 kb from any annotated genes. B) Genic regions containing at least one exon.

Many of CpG degenerations (GC to TG and GC to GA) within a gene are nonsynonymous changes and those CpG sites should be protected by purifying selection from degeneration if the gene is important for survival. In mammalian genomes, GC content at synonymous sites are also generally higher than that at intergenic non-coding sites [27]. We therefore hypothesized that the loss-of-function of macaque *CYP1A2 *(either completely or partially) may have initiated the degenerative substitutions at CpG sites in the gene. In order to test this hypothesis, we reanalyzed the polymorphism data of 24 *M. fascicularis *individuals in 27 genic (exons and adjacent non-coding sequences) and 27 intergenic regions (at least 100 kb away from any coding regions), which were randomly distributed on the autosomes and about 40 kb length in total [13]. As expected, the genic regions harbored more CpG sites per nucleotide than intergenic regions (*P *= 0.0047, Wilcoxon test). In Figure 3, nucleotide diversity and fraction of CpG sites are plotted for intergenic and genic regions. Statistically significant positive correlation (Spearman's *ρ *= 0.45, *P *= 0.020) was observed in the intergenic regions, while no significant correlation was observed in the genic regions (Spearman's *ρ *= -0.019, *P *= 0.93). The results indicate that the level of polymorphisms in intergenic regions is strongly affected by the number of CpG sites in those regions, whereas the functionality of genic regions prevents CpG site degeneration.

## Discussion

### Natural selection on drug-metabolizing genes

The process of drug metabolism has been under intense studies for biomedical benefits. Because humans are only natural organisms utilizing so-called "drugs", the impact of natural selection on drug metabolizing genes still remains unclear, except for some cases of insect herbivores specialized to their host plants [28,29]. Considering that many plant secondary metabolites are oxidized by CYP proteins and endogenous substrates controlling physiology such as hormones are also metabolized by CYP proteins, it is straightforward to access the effect of natural selection on the CYP protein diversity. For example, Thompson et al. proposed that the natural variation in human *CYP3A5 *is related to the retention of salt in a body and under natural selection according to latitudes [30]. A scan of recent selective sweeps in the human genome also detected a significant excess of *CYP *genes among those regions [31]. In macaques, Osada et al. found that *CYP3A5 *gene was highly differentiated between *M. fascicularis *and *M. mulatta *compared with other genes, suggesting the action of natural selection on the gene for local adaptation [13]. In the same study, they also found that *CYP1A2 *contained unusually high genetic diversity within the macaque species. Although the previous studies have revealed a weak hepatic expression of macaque *CYP1A2*, the weak expression does not necessary imply that macaque *CYP1A2 *is not biologically important. The main object of this study is elucidating the mode of natural selection on macaque *CYP1A2*.

### Gene conversion between *CYP1A *genes and effect on substrate specificity

Because many factors, including gene conversion between paralogous genes, could create highly polymorphic genes, we first clarified the pattern of molecular evolution of the CYP1A subfamily. Whether *CYP1A *subfamily may have expanded or contracted many times through the process of birth-and-death evolution of gene families [32], is an interesting question. For example, in *CYP2D *subfamily, lineage-specific gene losses in primates were reported [33], but the subfamily also experienced a large-scale expansion in non-primate placental mammals. We found that many vertebrate species genomes encoded *CYP1A1 *and *CYP1A2 *with the same head-to-head orientation as the human genome, and macaques did not harbor any other paralogous *CYP1A *genes created by recent gene duplication. A new method combining a window plot and maximum likelihood successfully detected the signature of gene conversion at the first 500 bp of exon 2 and entire exon 5, which correspond to SRS1 and SRS5, respectively. Our method is not sensitive to gene conversion tracts much shorter than the window size (150 bp), but successfully detected the gene conversion events in those regions. In addition, we did not find any signature of gene conversion using human and marmoset genes, indicating the last gene conversion event occurred before the divergence of *Catarrhini *and *Platyrrhini*. Because gene conversion is supposed to work effectively on young duplicates [34], we expect the two paralogs in primates continue diverging since gene conversion ceased. Interestingly, one amino acid at position 382 in SRS5 is responsible for substrate specificity of *CYP1A1/2 *gene to 7-ethoxyresorufin (382V) and 7-ethoxyresorufin (382L) [35]. The last gene conversion homogenized SRS5 of CYP1A1 and CYP1A2 and the substrate specificity should have been acquired after the event. Indeed, both macaque CYP1A1 and CYP1A2 proteins have 382V, but orangutan has 382V in CYP1A1 and 382L in CYP1A2, indicating that the mutation from valine to leucine that modified the substrate specificity fixed after the divergence of human and macaque lineages and before the divergence of human and orangutan lineages. In contrast to exon 2 and 5, the other exons showed much higher paralogous divergence. The regional heterogeneity of gene conversion pattern may indicate natural selection diversifying the substrate specificities of CYP1A1 and CYP1A2 has been maintaining the high paralogous divergence in those conversion-repressed regions, as shown in the cases of *Drosophila *transporter genes and yeast heat shock protein genes [36,37].

### *CYP1A2 *gene is dispensable in macaques

In contrast to our initial expectation, we found many null alleles were segregating both in *M. fascicularis *and *M. mulatta CYP1A2*. The highest allele frequency of these null alleles was 39% at site 1066 in *M. mulatta *(Table 3). Although we previously identified two null alleles in macaque *CYP3A4 *and *CYP3A5*, the null allele frequencies were less than 1% [38], indicating the null variants in *CYP3A4/5 *are strongly selected against by natural selection in contrast to *CYP1A2*. Because all *M. fascicularis *so far examined had weak gene expression of *CYP1A2*, mutations in regulatory regions should have become fixed in the common ancestors of macaques. However, it remains unclear whether the initial loss of gene expression was driven by positive selection under the "less is more" hypothesis [39], or due to the fixation of neutral or slightly deleterious alleles.

### Mechanisms of rapid decay of CpG sites

If macaque *CYP1A2 *gene is losing its function, why do we observe the high genetic diversity in populations? Although loss-of-function generally results in evolutionary patterns similar to patterns under neutrality, the genetic diversity in macaque *CYP1A2 *was significantly higher than *CYP1A1*, and much higher than the previously investigated background loci [13]. We found that primate *CYP1A2 *genes contained a considerable number of CpG sites, which are thought to mutate at much higher rates than other sites.

A number of mammalian genome studies have revealed that CpG sites are enriched in genic regions not only at nonsynonymous sites such as encoding arginine, but also at synonymous sites and introns. The reason why genic regions in mammalian genomes are enriched with CpG sites is not totally clear, but we could raise two hypotheses. One is the natural selection hypothesis, where natural selection is maintaining CpG sites within genes for the control of transcriptional regulation or integrity of genomes [40]. Another is the substitution bias hypothesis, where biased gene conversion is maintaining high GC content in genic regions [41] because of the association between recombination and gene density [42]. In particular, it was proposed that inter-locus gene conversion among multi-gene family members could increase CG content of those genes [43]. Although the latter mechanism would be important for creating the high GC regions in genomes, it cannot explain the deficiency of CpG degeneration in genic regions (Figure 3B), because under the substitution bias hypothesis we should observe many gain of CG as well as loss of CpG in genic regions. Therefore, at some extent, purifying selection protecting CpG sites within genic regions must play an essential role for maintaining CpG sites.

There are two ways how natural selection is protecting CpG sites from degeneration. Firstly, natural selection could keep CpG sites as unmethylated status in germ line cells, which can be observed in CpG-island associated genes. Secondly, CpG sites are methylated in germ line cells and tend to degenerate, but the degenerated alleles are removed from populations by purifying selection. Unfortunately, we could not determine which scenario is more plausible in the case of macaque *CYP1A2 *gene. Future studies investigating the methylation pattern within human and macaque *CYP1A *genes in many tissues would answer such questions. With either scenario, we could predict that once genes with many CpG sites lose its function, those genes would start to accumulate many mutations caused by rapid CpG degeneration.

### Effect of CpG site degeneration at species divergence level

In this study, our main focus was the effect of CpG degeneration on genetic diversity within species. However, the same caution should be taken in analyses between two diverged species, as suggested by Subramanian and Kumar [44]. As an extreme case, imagine a gene encoding many arginine residues. If the gene lost its function and those sites are methylated, many CpG sites start to degenerate and we would observe the acceleration of nonsynonymous changes relative to synonymous changes. Such pattern is often interpreted as the signature of positive selection, but we need to be careful for interpreting such data [45-47]. Indeed, when we compare the coding sequences of human and *M. fascicularis *reference *CYP1A2 *sequences, the ratio of non-synonymous substitution rate to synonymous substitution rate was 0.445, which was nearly two times higher than the genome-wide average [48], indicating that, although weakly, CpG degeneration affects the evolution rate of *CYP1A2 *at the apparent species divergence level.

## Conclusions

Our molecular evolution and population genetic study strongly supports that macaque CYP1A2 is no longer essential for *M. fascicularis *and *M. mulatta*. At some time after the divergence between humans and macaques, the major hepatic *CYP1A *gene was changed in the lineage to macaques. However, *CYP1A1 *gene expression pattern in macaques has not been completely switched to the human *CYP1A2 *expression pattern; the expression of macaque *CYP1A1 *is not so abundant without induction, but strongly induced by omeprazole, a CYP inducer [49,50]. The alteration of gene expression pattern could be possible by changes in the gene regulatory region, because *CYP1A1 *and *CYP1A2 *are aligned in a head-to-head orientation and shared a promoter region [4]. At this moment, we do not have any hypothesis explaining why the change of expression pattern and loss of function occurred only in the macaque lineage. Since the last gene conversion events between *CYP1A1 *and *CYP1A2 *occurred before the divergence between *Catarrhini *and *Platyrrhini*, the resurrection of function by gene conversion cannot explain the macaque-specific gene loss. Because the functional importance of *CYP1A1/2 *genes is highly different between humans and macaques, we have to be cautious in extrapolating a drug-testing data using substrates metabolized by *CYP1A *genes from macaques to humans, despite of their somewhat overlapping substrate specificities.

## Methods

### Sequence analysis

DNA sequences of vertebrate *CYP1A1/2 *genes were retrieved from a public database, and analyzed using MEGA 5 [51]. DDBJ/EMBL/Genbank accession numbers of analyzed cDNA sequences are as follows: macaque *CYP1A1 *(D17575), macaque *CYP1A2 *(D86474), mouse *CYP1A1 *(AK005000), mouse *CYP1A2 *(BC018298), rat *CYP1A1 *(X00469), rat *CYP1A2 *(BC127476), chicken *CYP1A4 *(X99453), chicken *CYP1A5 *(X99454), and medaka *CYP1A *(AY297923). The nucleotide sequences were aligned using the MUSCLE with default parameter setting using the information of amino acid sequences [52]. The phylogenetic tree of *CYP1A1/2 *was constructed using the amino acid sequences with the Dayhoff's distance matrix and the bootstrap resampling was performed 1000 times.

### Test of gene conversion

Quartet sequences (two paralogous genes from two different species) were used to detect the molecular evolution pattern under gene conversion. The nucleotide sequence alignments of exon 2, 3, and 7 were binned into 150-bp-length windows with 9 bp incremental steps. Because the nucleotide sequence alignments of exon 4, 5, and 6 were shorter than 150 bp, they were analyzed as single windows. For each window, we estimated the log-likelihood values of the type-N and type-C trees using PAML [22].

### Resequencing of macaque CYP1A1/2 genes

Whole blood was collected from 63 cynomolgus macaques (31 from Indochina and 32 from Indonesia, 3-8 years of age, 3-5 kg) and 28 rhesus macaques (from China, 2-3 years of age, weighing 3-5 kg). Genomic DNA was prepared from these blood samples using Gentra Puregene Blood kit (Qiagen, Valencia, CA) according to the manufacturer's instructions. The study was reviewed and approved by the Institutional Animal Care and Use Committee of Shin Nippon Biomedical Laboratories, Ltd (Kainan, Japan). Polymorphisms were identified by PCR amplification, followed by direct-sequencing. Briefly, PCR was performed in a 20 μL reaction containing 1 ng of genomic DNA, 5 pmole of each primer, and 1 unit of AmpliTaq Gold DNA polymerase (Applied Biosystems, Foster City, CA) using a thermal cycler (Applied Biosystems). For exons 2-5 of *CYP1A1*, EX Taq HS DNA polymerase (Takara, Tokyo, Japan) was used. Thermal cycler condition included an initial denaturation at 95°C for 10 min: 30 cycles of 20 s at 95°C, 30 s at 55-60°C, and 1 min at 72°C, followed by a final extension step of 5 min at 72°C. PCR products were sequenced using an ABI PRISM BigDye Terminator v3.0 Ready Reaction Cycle Sequencing Kit (Applied Biosystems), followed by electrophoresis on an ABI PRISM 3730 DNA Analyzer (Applied Biosystems). PCR/sequencing primers and annealing temperatures for PCR are listed in Table S1 (Additional File 1).

## Software Used

DNA sequences of *CYP1A1/2 *genes in vertebrate species were analyzed using MEGA 5 [51]. Sequence data were analyzed using the Sequencher software (Gene Codes, Ann Arbor, MI). Polymorphism data were analyzed using DnaSP 5.0 [53].

## List of abbreviations

CYP: cytochrome oxidase P450; SNP: single nucleotide polymorphism.

## Authors' contributions

NO and YU designed the research. YU carried out the experiments. NO and YU analyzed the data and wrote the paper. Both authors have read and approved the final manuscript.

## Supplementary Material

Additional file 1 **Table S1**. Primers used for PCR and sequencing. Click here for file
