# Supplementary material for: CpG site degeneration triggered by the loss of functional constraint created a highly polymorphic macaque drug-metabolizing gene, CYP1A2
Source: BMC Evol Biol. 2011 Oct 1;11:283. doi: 10.1186/1471-2148-11-283 (PMC3199271; doi:10.1186/1471-2148-11-283)
Supplement: Additional file 1 — Table S1. Primers used for PCR and sequencing. [file 1471-2148-11-283-S1.PDF]

Table S1. Primers used for PCR and sequencing.

Exons		Sequence (5′ -> 3′)	Annealing (°C)
CYP1A1			
1	F	CTCAACAACTTCTCCCCTCCT	58
	R	CCATACAGTTCCTCTTACCTCTGAC	
	S	AGGGCGATGATTTCAAGG	
	S	AACTTTTCAGGCCATTCTGG	
	S	CGCCAGGGTTTTCCCAGTCACGAC	
	S	AGCGGATAACAATTTCACACAGGAAAC	
2–5	F	GAGCACATCCAGGTGATAACAGTA	55
	R	ACCAGAGGAAGACAACTGAAGTG	
	S	GCTAGGAATAGTGAAGGACCAGAC	
	S	CTTACCTGTGGGGGATGGT	
	S	GGATAGAGGACAGGCAAGCA	
6	F	TCAGAGGTGCAGAGGAAAGG	58
	R	ACAGTAAAACCCCACACAGAATG	
	S	TTGCATTGATCCTCCTGTCC	
	S	CAGAGGCAAGTCCAGGGTAG	
CYP1A2			
1	F	GGCACAGGACACATGGTAGAT	58
	R	CCCAGAGAAGGGGAAGAGTT	
	S	GCCAAGCACAGAACACACAT	
	S	TGAGGTATCCAGAGCCTTCCTA	
2–4	FS	TGCTTCCCTGTGTTCACACTAAC	58
	R	TGAGAAGCCAGGAAGAGGAA	
	S	TAGCTTTCTCTCGCCCTAGC	
5	F	AGTTAAAGAACAGGAGGCAAGGAC	58
	RS	ATCCTGGTTGATTCCTGTGC	
	S	CTCTACCCCTTCCCTGTTCC	
6	F	TCCTTCCCACTTACCCTTCA	60
	R	GAGGAGAAACAAGGGCTGAGT	
	S	ACTTGTGCCTCAACAGAAGTCTC	
	S	GCTCAAATGATCCTCCAACCT	

F, forward primer; R, reverse primer; S, sequence primer.
